# Supplementary material for: Effect of the time to antivenom administration on recovery from snakebite envenoming-related coagulopathy in French Guiana
Source: PLoS Negl Trop Dis. 2023 Apr 24;17(4):e0011242. doi: 10.1371/journal.pntd.0011242 (PMC10159357; doi:10.1371/journal.pntd.0011242)
Supplement: Attachment — Submitted filename: Response to Nithya Venkatesan.docx [file pntd.0011242.s002.docx]

Dear Nithya Venkatesan,

According to your email, we confirm that the CRediT author contributions listed for every co-author are completed accurately and in full.

Our data are available from the Medical Information department of our hospital which is a non-author institutional point of contact. The e-mail to join the Medical Information department of Cayenne hospital is : [dim@ch-cayenne.fr](mailto:dim@ch-cayenne.fr)

1. We provided an Author Summary (184 words).
2. We removed all instances of trademark/copyright symbols throughout the text, including Tri® on line no 28, 89,124,125, AntivipmynTri® on line no 132, Tri® on line no 352.
3. We uploaded all main figures (Figure1 to Figure 5) as separate Figure files in .tif format.
4. We added in the ethics statement:

" The hospital’s institutional review board **and the Cayenne hospital ethics committee** approved …”

“**Formal** verbal consent was obtained from all patients or **parent/guardian** (when patients are <18 years or unable to consent) … ".

5. For Figure 1, we drew the image by hand from:

<https://d-maps.com/carte.php?num_car=15471&lang=en>

In the “Terms and conditions of use” in the website, it is stated that:

All the maps in this website are copyright protected.

They are modifiable, free for any use - even commercial - under the following conditions:

-The exact URL where the original map comes from must be mentioned

-The number of maps is limited to 10 per publication (website, DVD, Book...)

**We added in the legend of Figure 1:**

The original map from: <https://d-maps.com/carte.php?num_car=15471&lang=en>
