# Supplementary material for: Effect of the time to antivenom administration on recovery from snakebite envenoming-related coagulopathy in French Guiana
Source: PLoS Negl Trop Dis. 2023 Apr 24;17(4):e0011242. doi: 10.1371/journal.pntd.0011242 (PMC10159357; doi:10.1371/journal.pntd.0011242)
Supplement: Attachment — Submitted filename: Responses to reviewers comments.docx [file pntd.0011242.s003.docx]

Dear Editor,

We are grateful for your interest in our work and overall positive appreciation.

Please find enclosed the revised version of our article with a point per point response to the reviewer’s comments and a clean version with all changes included.

The number of page and line cited in response to reviewers refers to the numbers in the clean version.

----------------------------------------------------------------------------------------------------------------------------------

Responses to reviewers’ comments:

**Reviewer #1:** Methods:

Add more info regarding Antivipmyn Tri®. Type of antivenom (Fab?) and the snake venoms that it neutralizes. Add info also regarding number of recommended vials. Besides that, the methods are very detailed.

The following text was added (P5 L125-134):

Antivipmyn Tri® is a freeze-dried F(ab’)2 polyvalent antivenom produced by Instituto Bioclon, in Mexico—Mexico Registry N 58583 SSA IV. It Is prepared by immunizing horses with venoms of *Bothrops asper*, *Crotalus durissus terrificus*, *and Lachesis muta*. According to the manufacturer, it is indicated for the treatment of envenoming by vipers, such as *Bothrops atrox*, *Bothrops brazili, Bothrops asper, Bothrops neuwiedii, Bothrops alternatus, Bothrops jararacussu, Bothrops venezuelensis, Bothrops pictus, Crotalus durissus terrificus, Crotalus durissus durissus, Lachesis muta stenophrys, Lachesis muta muta, Sistrurus* spp., and *Agkistrodon* spp.

Reviewer #2: The study proposed an analysis of accidents that occurred in French Guiana with a difference between early and delayed 6-hour VA administration. This context is interesting since 6 hours is a gold standard for receiving VA and after this time the risk of complications gradually increases.

Regarding the classification of poisoning, the authors could describe how it is done according to the conclusions of the international symposium held in French Guiana in 2017 cited in the method to make it clearer.

The following text was added (P6 L146-151):

The grading system is based on three grades (I: mild, II: moderate, and III: severe) including a large number of signs and symptoms (Table 1).

Table 1: The grading system for classifying the severity of envenoming

|  |  | Grade | | |
| --- | --- | --- | --- | --- |
|  |  | I | II | III |
| Coagulation disorder* |  | **+** | **+** | **+** |
| Local signs | Pain | **+** | **+** | **+** |
|  | Swelling | Not exceeding  elbow or knee | Exceeding  elbow or knee | Beyond the  root of the limb |
|  | Blister | - | + | + |
|  | Necrosis | - | - | + |
| Local bleeding |  | - | + | + |
| Systemic bleeding |  | - | - | + |
| Systemic manifestations  (Hypotension, Renal failure, Coma, Respiratory failure) | | - | - | Organ failure |

*Based on results of the 20-min whole blood clotting test and laboratory analysis.

The following text was added (P6 L142-143): The culprit snake was identified based on the patient description, photographs, or on the physical examination of the captured snake.

The following text was added (P5 L132-134): The protocol of AntivipmynTri® used was already described (6 vials whatever the grade of envenoming), and its efficacy was already studied [2, 13].

2. Resiere D, Houcke S, Pujo JM, Mayence C, Mathien C, NkontCho F, Blaise N, Demar MP, Hommel D, Kallel H, (2020) Clinical Features and Management of Snakebite Envenoming in French Guiana. Toxins (Basel).

13. J-P Chippaux, (2002) Les envenimations ophidiennes en Guyane Française. Med Trop 62: 177–184

What is the maximum follow-up time for patients?

The following text was added (P7-8 L179-183): Patients were hospitalized in ICU until recovery of the coagulation disorders. Then they are transferred to the surgical ward and followed until day 8 from the bite or until recovery of the skin lesion when it takes more time to heal. After that, patients are followed in the outpatient clinic one week after the hospital discharge.

What were the patient exclusion criteria set at baseline?

The following text was added (P5 L118-121): We excluded all patients hospitalized for SB envenoming lasting more than 48 hours after the bite, those who did not receive antivenom, and those who did not present clinical or biological signs of envenoming.

What was the estimated number of cases in the period evaluated? Did the authors make a sample calculation?

In our study, the number of patients was obtained from the period outlined in the study. Indeed, during the study period, 198 patients were hospitalized for SB and 126 of them were included in our study. Our study was an observational and not interventional work. For this, there is no need for a sample calculation.

Did the laboratory tests have a frequency and interval period to be performed?

The following text was added (P7 L173-175): According to the hospital protocol, laboratory tests were performed every 6 hours from hospitalization until recovery of the coagulation parameters (fibrinogen >1.5 g/L).

**Reviewer #3:** This is an interesting study about the effect of the time to antivenom (AV) administration on recovery of the effects of snakebite envenoming in patients receiving AV early (in the first 6 hours after bite) or lately (6 hours or more after bite).

Objectives are clearly stated, and the study design is in accordance with the purposes. It is not described how the sample size was defined. It may be inferred that number of patients was obtained from the period outlined in the study. However, for some not frequent outcomes, sample size may not have been sufficient to show statistical significance in the comparison between groups, such as necrosis, blisters and dialysis. Authors indicate this limitation, suggesting the possibility of confounding factors, but do not explain which ones. Readers would benefit from greater detail in this respect.

In our study, the number of patients was obtained from the period outlined in the study. Indeed, during the study period, 198 patients were hospitalized for SB and 126 of them were included in our study. Our study was an observational and not interventional work. For this, there is no need for a sample calculation.

We agree that for some not frequent outcomes, sample size may not have been sufficient to show statistical significance in the comparison between groups, such as necrosis, blisters and dialysis. This issue was indicated in the limitation section.

The following text was added (P22 L476-478): Indeed, in some not frequent outcomes, sample size may not be sufficient to show statistical significance in the comparison between groups.

We agree that the limitation is about the sample size mainly in the severe patient’s group and not about confounders. May be the only confounders are venom related skin symptoms which can be confounded with inflammatory symptoms when the envenomation turns to infection.

We deleted “ … without excluding unmeasured confounders” from the sentence.

----------------------------------------------------------------------------------------------------------------------------------

**Reviewer #1:** The results are fine.

**Reviewer #2:** In line 206 the authors described those 52 patients had the snake identified, was it by photo or did they bring the snake? 51 snakes were Bothrops atrox and what was the other 1 species?

The following text was added (P6 L142-143): The culprit snake was identified based on the patient description, photographs, or on the physical examination of the captured snake.

The 52th snake was a *Bothrops bilineatus*. It was a 9 years girl bitten on the face.

The following text was added (P10 L238): “It was *Bothrops atrox* in 51 cases and *B. bilineatus* in one case.”

Line 211 Do the authors describe the main signs and symptoms of the patients, is this information from the patient's arrival at the hospital or at another time? The clinical information is in general, wouldn't it be better to divide into the group of early AV and delayed AV as in the rest of the results?

The reported signs and symptoms are those recorded at hospital admission. The progression of the symptoms is described in table 1 under the subtitle “Worsening skin lesions”

The title of Table 1 was modified to “Epidemiological and clinical parameters at admission” and symptoms progression during hospitalization was indicated by “ # “ in the table.

The following text was added in the text (P11 L245-246): The only parameter showing a statistical difference between the two groups (early and delayed AV) was the local haemorrhage (24.4 vs. 5.4%, p=0.002).

Table 1 has the definition of GRADE I, II and III, please describe clearly in the methodology to improve understanding of this classification.

The following text and table were added in the Method section (P6 L146-151): The grading system for classifying the severity of envenoming is based on three grades (I: mild, II: moderate, III: severe) including a large number of signs and symptoms (Table 1).

Table 1: The grading system for classifying the severity of envenoming

|  |  | Grade | | |
| --- | --- | --- | --- | --- |
|  |  | I | II | III |
| Coagulation disorder |  | **+** | **+** | **+** |
| Local signs | Pain | **+** | **+** | **+** |
|  | Swelling | Not exceeding  elbow or knee | Exceeding  elbow or knee | Beyond the  root of the limb |
|  | Blister | - | + | + |
|  | Necrosis | - | - | + |
| Local bleeding |  | - | + | + |
| Systemic bleeding |  | - | - | + |
| Systemic manifestations  (Hypotension, Renal failure, Coma, Respiratory failure) | | - | - | Organ failure |

WBCT20: 20-min whole blood clotting test.

In table 3, not all patients underwent all laboratory tests, some of these, for example Factor X: out of 104, only 11 were evaluated in the table for Time from SB to normal and Time from AV to normal FX. Why was information not presented on the 104 patients who had this abnormal value? Thus, the small number of results would not be good for conclusions and analyzes due to possible biases. Is this the correct interpretation or am I mistaken?

Coagulation factors are dosed in all patients at admission. While, during the follow up, they are dosed only when the prothrombin time is <60%. In case of PT>60%, the coagulation factors are considered normal and then, are not dosed.

The following text was added (P7 L172-173): Coagulation factors are dosed only when the prothrombin time is less than 60%.

The fact of not having the monitoring of the abnormal exams of the 119 patients left me confused in the construction of figures 3 and 4, how many patients were considered to perform the graph calculations?

Figure 3: the construction of the graph was based on the results obtained from 29 and 47 patients for factors II and V. The construction regarding fibrinogen was based on the results obtained from 112 patients (only 7 patients are missing from the late AV group).

We added in the legend of the figure 3: The number of cases in whom the parameter was analyzed (Total, Early/Delayed) was: factor II (29, 13/16), factor V (47, 24/23), fibrinogen (112, 45/67).

Figure 4: the construction of the graph was based on the results obtained from 112 patients. 7 patients are missing from the late AV group, 4 of them because of hemolysis and difficulty of dosing.

We changed in the legend of the figure 4: SB and antivenom administration [≤6h (red line, n=45) vs. >6h (blue line, n=67)].

**Reviewer #3:** Statistical tests were correctly applied, but some numerical variables, such as fibrinogen and platelets, were not analyzed according to their medians. Authors preferred to convert them in discrete variables (number and percentage of patients with defibrinogenation, number and percentage of patients with thrombocytopenia). Would the comparison be different if variables se variables present different

Continuous data were analyzed regarding their value and were also converted to categorical data to indicate abnormality. Indeed, the main expected result is how much patients present abnormality regarding the studied parameter. For example, if we compare two groups of patients with a median/mean platelet count at 250 and 160 G/L with a significant difference. This result is not informative since the two values are normal. However, in this context, the needed information is not about the platelet count absolute value but about the number of patients with thrombocytopenia.

Rhabdomyolysis and hemolysis were included as variables to be studied, but their meaning in Bothrops envenoming in FG was not discussed. Laboratory tests were performed but values of CK (for rhabdomyolysis), LDH, bilirubin and haptologlobin (for hemolysis) were omitted. May these parameters did not show difference between groups, but authors should give some explanation.

The reported information is not about the absolute value of the parameter dosage but about the number of patients with the corresponding abnormality.

We added in the method section the normal range for LDH and haptoglobin according to our laboratory technique.

---------------------------------------------------------------------------------------------------------------------------------

**Reviewer #1:** Yes, the conclusion support the study.

**Reviewer #2:** Some parameters were associated in the statistical analysis, such as the first visit in a remote area, snake identification and local hemorrhage. However, only remote area care was well discussed, could the authors include the other points mentioned in the discussion as well?

The conclusion is a point per point response to the objectives of the study. Adding information about all statistically significant parameters can minimize the main message of the study. We prefer to keep the conclusions as they are in this version.

As for Factor V, aPTT, Defibrinogenation, I need clarification on the number of patients who completed the exams to issue a more correct opinion, as I was in doubt in interpreting the information in Table 3 and graphs 3 and 4.

The number of patients who completed the exams is indicated in the tables in the column “Nb”.

In the legend of figure 3 and 4 we added information about the number of patients who completed the exams:

We added in the legend of the figure 3: The number of cases in whom the parameter was analyzed (Total, Early/Delayed): factor II (29, 13/16), factor V (47, 24/23), fibrinogen (112, 45/67).

We changed in the legend of the figure 4: SB and antivenom administration [≤6h (red line, n=45) vs. >6h (blue line, n=67)].

Figure 5 and the explanation given in the text were very good, however, this would not be the appropriate place for this information. So, I suggest that this be in the methodology section.

Figure 5 represents a scheme of the proposed fibrinogen concentration kinetics following SB by *B. atrox*. This hypothesis is based on the results obtained in our study and is part of the discussion of our results. For this, we believe that the most appropriate place of this figure is in the discussion section.

In the limitation of the study, the authors describe that the entry of GRADE I patients that limited the evaluation of the time to the effects of VA, could the authors how this limited the study? Were the patients discharged early, not allowing the collection of exams and clinical evaluation to monitor the complete recovery of parameters, or was there another reason? Regarding the limitation of the identification of the snake in 47.3% of the cases - in line 207 43.7% were described - the authors could have a moment in the discussion that the stimulus of bringing and/or killing the snake should not be done in order not to increase the risk of new accidents and that the classification of the type of snake responsible for the envenomation is carried out by the patient's clinic, that is, this should not be considered a limitation of the study.

We added in the limitation section the following text (P22 L467-469): “Indeed, in some not frequent outcomes, sample size may not have been sufficient to show statistical significance in the comparison between groups.”

We agree that bringing and/or killing the snake should not be done in order not to increase the risk of new accidents.

The following text was added to the limitation section (P22 L479-481): Identification was based on the patient description, photographs, or on the physical examination of the captured snake. In this context, we recommend against bringing and/or killing the snake in order not to increase the risk of new accidents.

**Reviewer #3:** It is interesting the theory proposed to explain the higher percentage of patients with fibrinogen levels > 0.35 g/L in those who received AV late, compared to those treated early. Despite the correct recommendation for AV administration in any circumstance of systemic envenoming, could grade I patients suggest spontaneous recovery of coagulopathy in patients mildly envenomed?

Patients who are managed tardily have a fibrinogen dosage>0.35 g/L because the process of correcting coagulopathy has started. However, we believe that AV must be administered because the partial correction of fibrinogen did not indicate the complete recovery of the envenoming process.

The availability of antivenom in remote health facilities is strongly emphasized as a key aspect to reduce morbidity and mortality of snakebite envenoming in rural and traditional populations. This is an important contribution of study and could be complemented with some discussion regarding the type of health professional to be responsible for the administration of antivenom. Should be a medical doctor? In places where this type of professional is not available, could a nurse of health agent administer AV? What about the risk of complications of an adverse reaction to antivenom if used in a primary care unit?

This is a very interesting point since SB envenoming is one of the examples of inequality of access to care.

We added in the discussion section the following text (P19 L394-398): “European and French laws require that AV be administered by a nurse supervised by a doctor who can manage adverse reactions and anaphylactic shock. Thus, some French Guiana HCCs are planned to be provided by emergency doctors and trained nurses for AV administration and urgent snake bitten patients’ management.”

--------------------------------------------------------------------------------------------------------------------------------

**Reviewer #1:** Minor revision

**Reviewer #2:** The authors need to clarify the laboratory results.

Please see the explanations above

**Reviewer #3:** Line 139: "medical observation" is more appropriate than "surveillance"

We made the change

Table 1: correct typing "snake identification"

We made the change

Table 3: include legend, similarly to table 1 and 2.

We made the change

-----------------------------------------------------------------------------------------------------------------------------------

**Reviewer #1:** The article intitled Effect of the time to antivenom administration on recovery from snakebite envenoming- related coagulopathy in French Guiana is interesting. Although not novel (other studies have investigated the same) there is a lack of literature info regarding the snakebite treatment FG. The manuscript is easy to read; however, there are some grammar and other mistakes. I encourage authors read it again and have the article read by a native speaker as well. I really liked Figure 1, it is very explicative. For me the main results are in Figure 3. Is the group the pioneer to show that? Please check the literature and make it clear. Based on that, I also encourage a quick discussion regarding the hospital costs comparing the time the patient stay in the hospital (receiving early and late AV).

Previous studies have used Kaplan Mayer analysis and Error bars graphical representations to analyze the time difference to reach normal values of the coagulation parameters.

Regarding the cost, the length of hospital stay was 33% longer in patients receiving AV tardily 8 days (5 - 13) vs. 6 days (4 - 10), p=0.038. Accordingly, the cost was higher. However, since we did not attempt to carryout an analysis of the costs, we prefer not to include this point in our discussion. Further studies will focus on cost of treatment issues.

Minor issues:

Line 38, 43, 107, 208, etc: change hr to h (check all the manuscript)

We changed “hr” to “h” throughout the document

Lines 39-40: to correct laboratory coagulation parameters? Maybe to return to normal levels of coagulation. Please rewrite.

We made the change

Line 42: change sites to areas

We made the change

Line 43: change it to The time

We made the change

Line 58: remove comma before such

We made the change

Line 66: a loss of chance???? Please rewrite

We changed to “… consequently, lesser chances of envenomed victims”

Line 194: medevac???? See 320 too.

We changed to medical evacuation

Line 315: put together snakebite

We changed accordingly

Methods:

Add more info regarding Antivipmyn Tri®. Type of antivenom (Fab?) and the snake venoms that it neutralizes. Add info also regarding number of recommended vials.

This was added in the manuscript (see above)

**Reviewer #2:** The article is interesting mainly because it is a follow-up of patients, however some information is not clear to adequately respond to the objective of the study and understand the evolution of the clinical condition of the patients as described in the recommendations above.

**Reviewer #3:** (No Response)

----------------------------------------------------------------------------------------------------------------------------------

Dear Nithya Venkatesan,

According to your email, we confirm that the CRediT author contributions listed for every co-author are completed accurately and in full.

Our data are available from the Medical Information department of our hospital which is a non-author institutional point of contact. The e-mail to join the Medical Information department of Cayenne hospital is: [dim@ch-cayenne.fr](mailto:dim@ch-cayenne.fr)

1. We provided an Author Summary (184 words).
2. We removed all instances of trademark/copyright symbols throughout the text, including Tri® on line no 28, 89,124,125, AntivipmynTri® on line no 132, Tri® on line no 352.
3. We uploaded all main figures (Figure1 to Figure 5) as separate Figure files in .tif format. In addition, Figure files were uploaded to the Preflight Analysis and Conversion Engine (PACE) digital diagnostic tool to meet PLOS requirements.
4. We added in the ethics statement:

" The hospital’s institutional review board **and the Cayenne hospital ethics committee** approved …”

“**Formal** verbal consent was obtained from all patients or **parent/guardian** (when patients are <18 years or unable to consent) … ".

5. For Figure 1, we drew the image by hand from:

<https://d-maps.com/carte.php?num_car=15471&lang=en>

In the “Terms and conditions of use” in the website, it is stated that:

All the maps in this website are copyright protected.

They are modifiable, free for any use - even commercial - under the following conditions:

-The exact URL where the original map comes from must be mentioned

-The number of maps is limited to 10 per publication (website, DVD, Book...)

**We added in the legend of Figure 1:**

The original map from: <https://d-maps.com/carte.php?num_car=15471&lang=en>

Figure Files:

While revising your submission, please upload your figure files to the Preflight Analysis and Conversion Engine (PACE) digital diagnostic tool, https://pacev2.apexcovantage.com. PACE helps ensure that figures meet PLOS requirements. To use PACE, you must first register as a user. Then, login and navigate to the UPLOAD tab, where you will find detailed instructions on how to use the tool. If you encounter any issues or have any questions when using PACE, please email us at [figures@plos.org.](mailto:figures@plos.org.)

Figure files were uploaded to the Preflight Analysis and Conversion Engine (PACE) digital diagnostic tool to meet PLOS requirements.

Data Requirements:

Please note that, as a condition of publication, PLOS' data policy requires that you make available all data used to draw the conclusions outlined in your manuscript. Data must be deposited in an appropriate repository, included within the body of the manuscript, or uploaded as supporting information. This includes all numerical values that were used to generate graphs, histograms etc.. For an example see here: http://www.plosbiology.org/article/info%3Adoi%2F10.1371%2Fjournal.pbio.1001908#s5.

All data used to draw the conclusions outlined in our manuscript are deposited in the clinical investigation center ([publication.recherche@ch-cayenne.fr](mailto:publication.recherche@ch-cayenne.fr)) and the medical information department ([dim@ch-cayenne.fr](mailto:dim@ch-cayenne.fr)) of the Cayenne Hospital. They are available upon request from these two hospital repositories.

Reproducibility:

To enhance the reproducibility of your results, we recommend that you deposit your laboratory protocols in protocols.io, where a protocol can be assigned its own identifier (DOI) such that it can be cited independently in the future. Additionally, PLOS ONE offers an option to publish peer-reviewed clinical study protocols. Read more information on sharing protocols at <https://plos.org/protocols?utm_medium=editorial-email&utm_source=authorletters&utm_campaign=protocols>

Our study is observational non internetional work. It is based on data collection and statistical analysis as detailed in the method section. Accordingly, there was no specific clinical or laboratory protocol.
